# Supplementary material for: BREM-SC: a bayesian random effects mixture model for joint clustering single cell multi-omics data
Source: Nucleic Acids Res. 2020 May 7;48(11):5814–24. doi: 10.1093/nar/gkaa314 (PMC7293045; doi:10.1093/nar/gkaa314)
Supplement: gkaa314_Supplemental_File [file gkaa314_supplemental_file.pdf]

## SUPPLEMENTARY DATA

### Supplementary Methods - Details of Gibbs Sampling

We proposed a general Bayesian framework for estimation. We used Gibbs sample to iteratively update $z_j$, $\alpha_{i(k)}^{(1)}$, $\alpha_{d(k)}^{(2)}$ and $b_j$. Specifically, we used random walk Metropolis within Gibbs to iteratively update $b_j$, $\alpha_{i(k)}^{(1)}$ and $\alpha_{d(k)}^{(2)}$.

For a given cell $j$, the conditional distribution for $z_j$ follows a Multinomial distribution, where

$$P(z_j = k| \dots) = \frac{1}{constant}\left(\prod_{i=1}^{G}\frac{\Gamma\left(x_{ij}^{(1)} + \alpha_{i(k)}^{(1)}b_j\right)}{\Gamma\left(\alpha_{i(k)}^{(1)}b_j\right)}\right)\frac{\Gamma\left(|\boldsymbol{\alpha}_{(\boldsymbol{k})}^{(\boldsymbol{1})}b_j|\right)}{\Gamma\left(T_j^{(1)} + |\boldsymbol{\alpha}_{(\boldsymbol{k})}^{(\boldsymbol{1})}b_j|\right)}$$

$$\left(\prod_{d=1}^{D}\frac{\Gamma\left(x_{dj}^{(2)}+\alpha_{d(k)}^{(2)}b_j\right)}{\Gamma\left(\alpha_{d(k)}^{(2)}b_j\right)}\right)\frac{\Gamma(|\boldsymbol{\alpha}_{(\boldsymbol{k})}^{(\boldsymbol{2})}b_j|)}{\Gamma(T_j^{(2)}+|\boldsymbol{\alpha}_{(\boldsymbol{k})}^{(\boldsymbol{2})}b_j|)}\pi_k.$$

where the normalization constant is:

$$\sum_{k=1}^{K}\left\{\left(\prod_{i=1}^{G}\frac{\Gamma\left(x_{ij}^{(1)}+\alpha_{i(k)}^{(1)}b_j\right)}{\Gamma\left(\alpha_{i(k)}^{(1)}b_j\right)}\right)\frac{\Gamma(|\boldsymbol{\alpha}_{(\boldsymbol{k})}^{(\boldsymbol{1})}b_j|)}{\Gamma(T_j^{(1)}+|\boldsymbol{\alpha}_{(\boldsymbol{k})}^{(\boldsymbol{1})}b_j|)}\left(\prod_{d=1}^{D}\frac{\Gamma\left(x_{dj}^{(2)}+\alpha_{d(k)}^{(2)}b_j\right)}{\Gamma\left(\alpha_{d(k)}^{(2)}b_j\right)}\right)\frac{\Gamma(|\boldsymbol{\alpha}_{(\boldsymbol{k})}^{(\boldsymbol{2})}b_j|)}{\Gamma(T_j^{(2)}+|\boldsymbol{\alpha}_{(\boldsymbol{k})}^{(\boldsymbol{2})}b_j|)}\pi_k\right\}.$$

For a given gene $i$ and cell type $k$, the conditional log likelihood for $\alpha_{i(k)}^{(1)}$ is

$$logP\left(\alpha_{i(k)}^{(1)}\middle| \dots\right) \propto \sum_{j=1}^{C} I(z_j = k)\log\left\{\left(\frac{\Gamma\left(x_{ij}^{(1)}+\alpha_{i(k)}^{(1)}b_j\right)}{\Gamma\left(\alpha_{i(k)}^{(1)}b_j\right)}\right)\frac{\Gamma(|\boldsymbol{\alpha}_{(\boldsymbol{k})}^{(\boldsymbol{1})}b_j|)}{\Gamma(T_j^{(1)}+|\boldsymbol{\alpha}_{(\boldsymbol{k})}^{(\boldsymbol{1})}b_j|)}\right\}.$$

Similarly, for a given ADT marker $d$ and cell type $k$, the conditional log likelihood for $\alpha_{d(k)}^{(2)}$ is

$$logP\left(\alpha_{d(k)}^{(2)}\middle| \dots\right) \propto \sum_{j=1}^{C} I(z_j = k)\log\left\{\left(\frac{\Gamma\left(x_{dj}^{(2)}+\alpha_{d(k)}^{(2)}b_j\right)}{\Gamma\left(\alpha_{d(k)}^{(2)}b_j\right)}\right)\frac{\Gamma(|\boldsymbol{\alpha}_{(\boldsymbol{k})}^{(\boldsymbol{2})}b_j|)}{\Gamma(T_j^{(2)}+|\boldsymbol{\alpha}_{(\boldsymbol{k})}^{(\boldsymbol{2})}b_j|)}\right\}.$$

Finally, for a given cell $j$, we have the conditional log likelihood for $b_j$ as:

$$logP(b_j| \dots) \propto \sum_{k=1}^{K} I(z_j = k))\ log$$

$$\left\{\left(\prod_{i=1}^{G}\frac{\Gamma\left(x_{ij}^{(1)}+\alpha_{i(k)}^{(1)}b_j\right)}{\Gamma\left(\alpha_{i(k)}^{(1)}b_j\right)}\right)\frac{\Gamma\left(|\boldsymbol{\alpha}_{(\boldsymbol{k})}^{(\boldsymbol{1})}b_j|\right)}{\Gamma\left(T_j^{(1)}+|\boldsymbol{\alpha}_{(\boldsymbol{k})}^{(\boldsymbol{1})}b_j|\right)}\left(\prod_{d=1}^{D}\frac{\Gamma\left(x_{dj}^{(2)}+\alpha_{d(k)}^{(2)}b_j\right)}{\Gamma\left(\alpha_{d(k)}^{(2)}b_j\right)}\right)\frac{\Gamma\left(|\boldsymbol{\alpha}_{(\boldsymbol{k})}^{(\boldsymbol{2})}b_j|\right)}{\Gamma\left(T_j^{(2)}+|\boldsymbol{\alpha}_{(\boldsymbol{k})}^{(\boldsymbol{2})}b_j|\right)}\right\} -logb_j - \frac{(logb_j)^2}{2\sigma_b^2}.$$

**Table S1.** Computational speed and memory consumption benchmark for BREM-SC on one computing thread (with 200/500/1000 MCMCs)

| | 200 MCMCs | 500 MCMCs | 1000 MCMCs | Max Memory Usage |
|---|---|---|---|---|
| 1200 total number of cells* | | | | |
| | 200 MCMCs | 500 MCMCs | 1000 MCMCs | Max Memory Usage |
| 100 Genes | 0.8 min | 2.2 min | 5.2 min | 112.3 M |
| 500 Genes | 4.3 min | 10.8 min | 20.9 min | 136.3 M |
| 1000 Genes | 8.5 min | 20.6 min | 33.8 min | 200.1 M |
| 4800 total number of cells* | | | | |
| | 200 MCMCs | 500 MCMCs | 1000 MCMCs | Max Memory Usage |
| 100 Genes | 3.9 min | 9.3 min | 18.3 min | 139.2 M |
| 500 Genes | 15.3 min | 33.9 min | 60.8 min | 278.3 M |
| 1000 Genes | 30.27 min | 60.3 min | 118.7 min | 414.2 M |

* The number of clusters is set at 4; the number of protein markers is set at 20.

**Table S2.** Performance (ARI) of jointDIMM-SC compared to BREM-SC on two real CITE-seq datasets

| | ARI<br>Mean (SD), [Range] | AMI<br>Mean (SD), [Range] |
|---|---|---|
| Public human PBMC ($\widehat{\sigma_b} = 0.7$) | | |
| BREM-SC | 0.728 (0.091), [0.585, 0.840] | 0.737 (0.048), [0.674, 0.800] |
| jointDIMM-SC | 0.711 (0.104), [0.529, 0.836] | 0.723 (0.060), [0.637, 0.811] |
| In-house human PBMC ($\widehat{\sigma_b} = 1.2$) | | |
| BREM-SC | 0.966 (0.025), [0.917, 0.985] | 0.944 (0.039), [0.869, 0.970] |
| jointDIMM-SC | 0.928 (0.073), [0.748, 0.993] | 0.904 (0.062), [0.809, 0.976] |

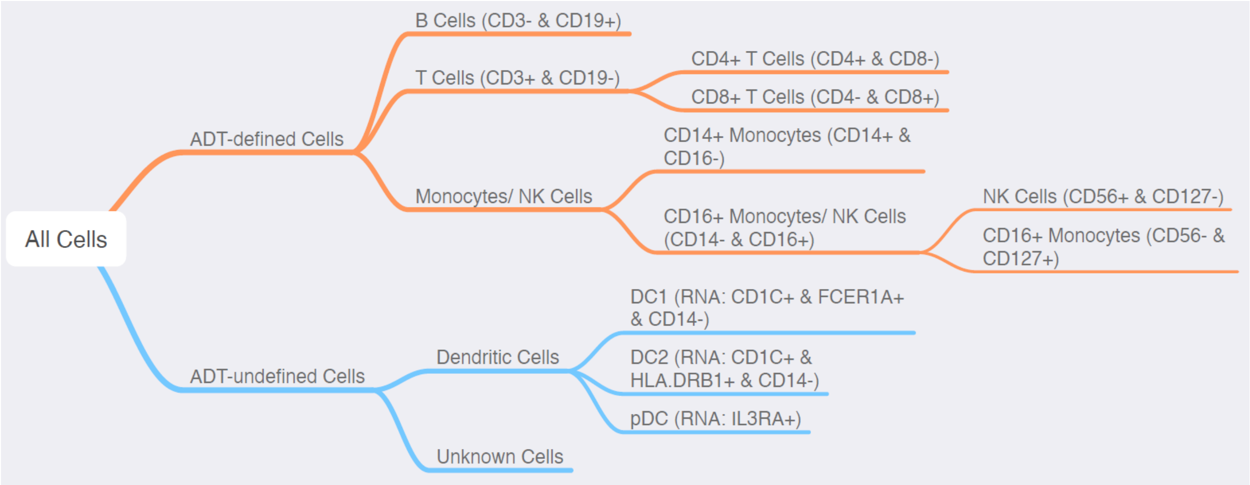


**Figure S1.** General workflow illustrating how to get the approximated truth based on ADT and RNA markers. For the public human PBMC dataset, both ADT and RNA markers are used. For the in-house dataset, only ADT markers are used.

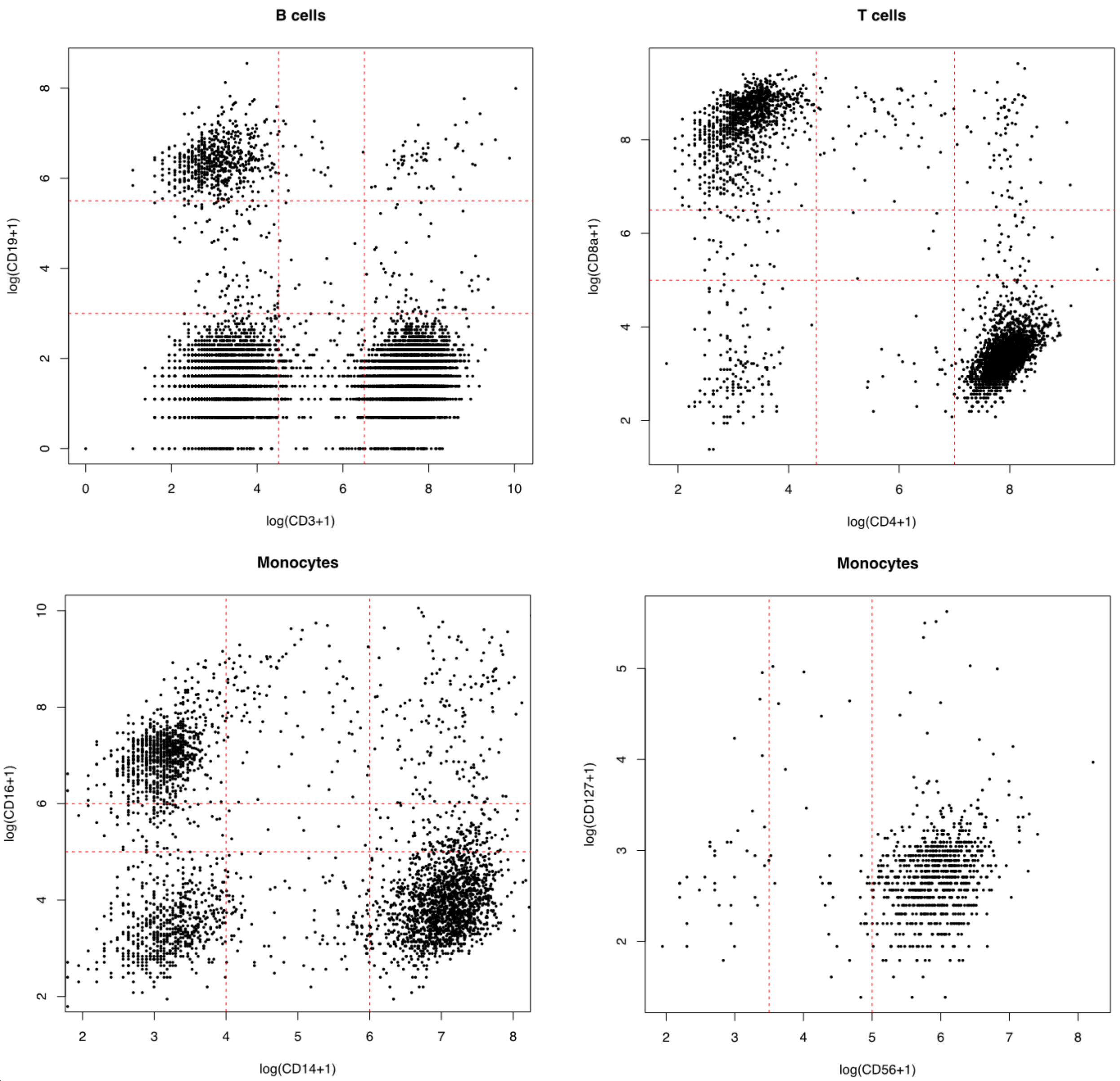


**Figure S2.** Scatter plot of cells illustrating how to get the approximated truth in 10X public human PBMC dataset.

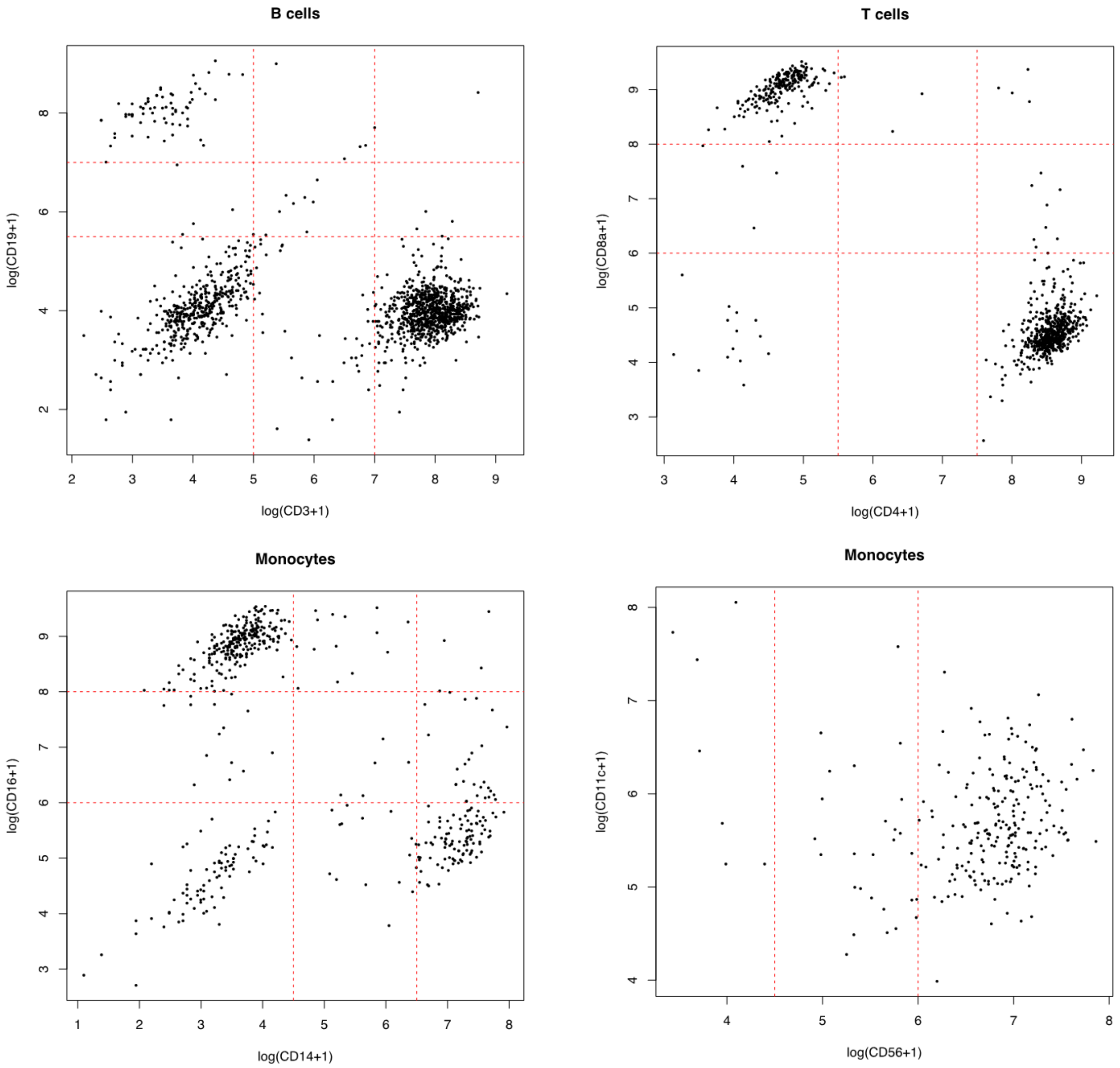


**Figure S3.** Scatter plot of cells illustrating how to get the approximated truth for in-house human PBMC dataset

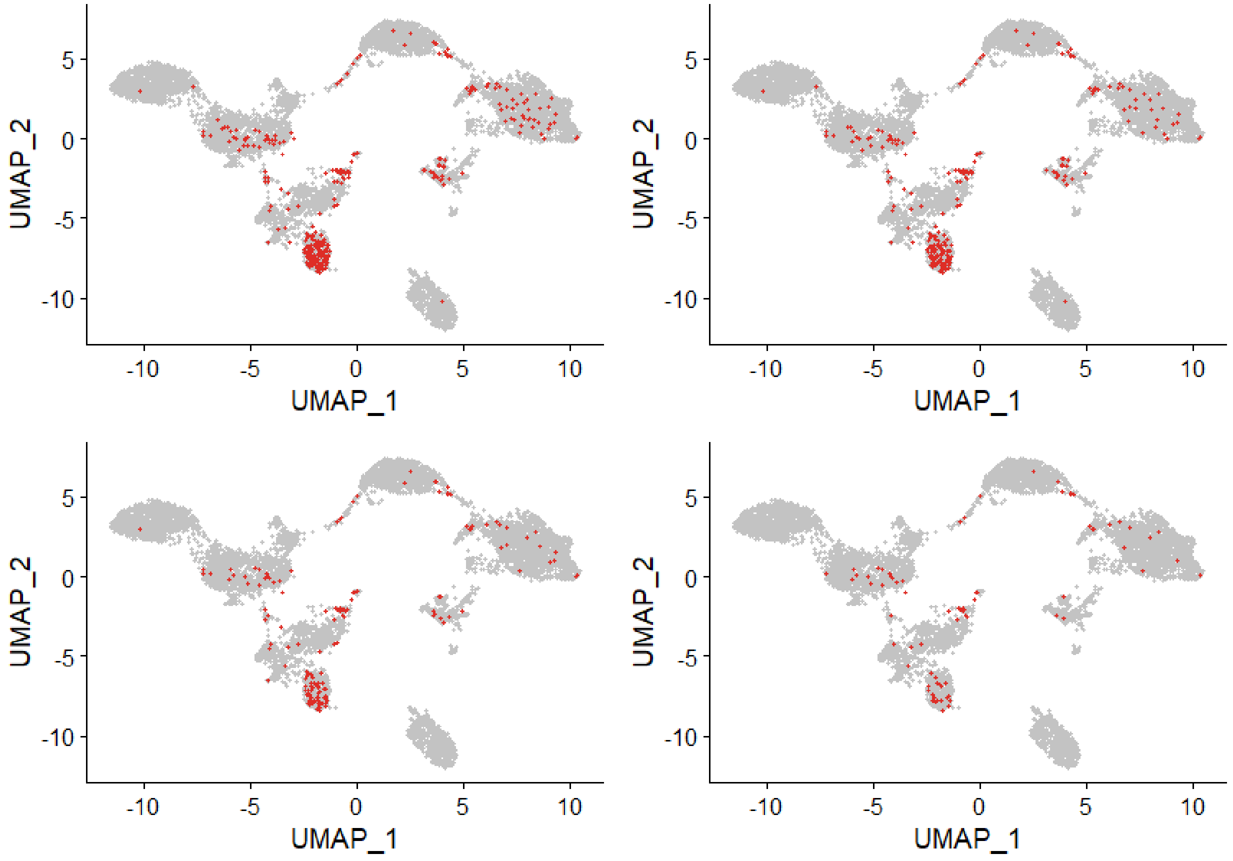


**Figure S4.** Illustration of "soft clustering" property by highlighting "vague" cells (4%, top left; 3% top right; 2% bottom left; 1% bottom right) with the lowest certainty from BREM-SC.

S5A

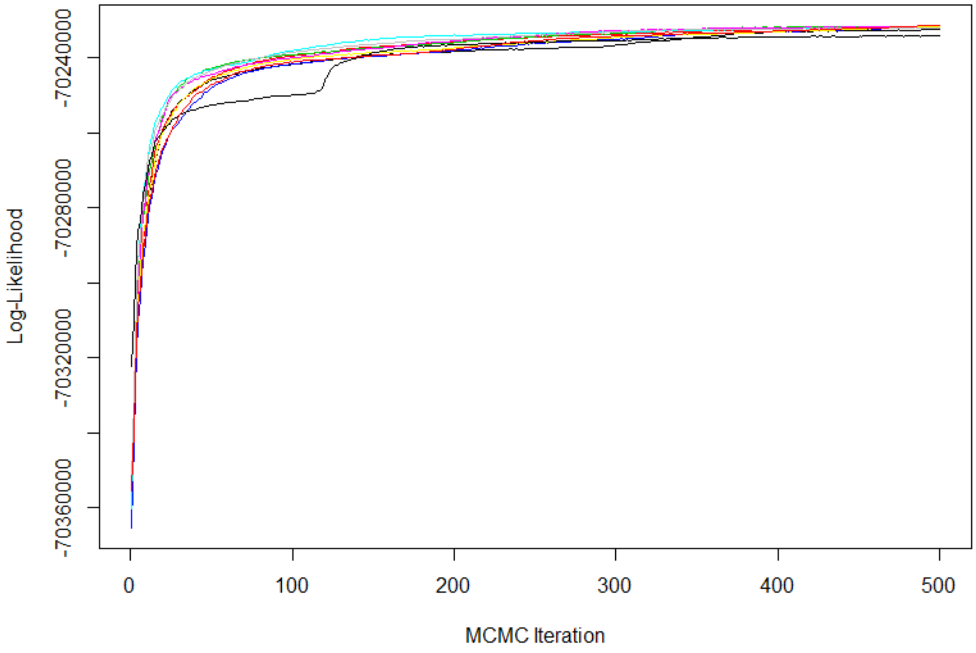


S5B

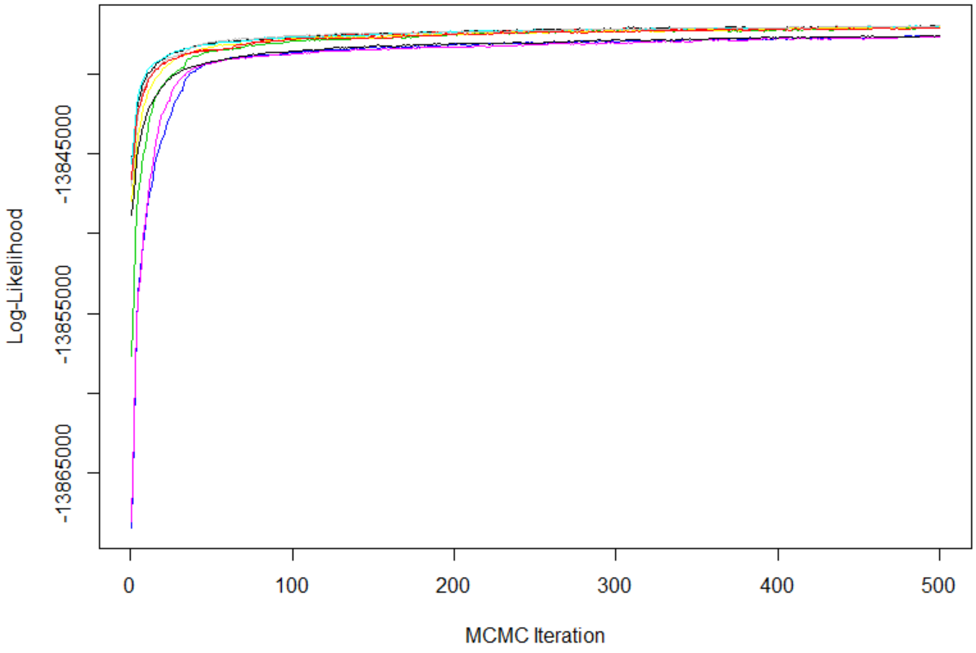


**Figure S5.** Convergence of MCMC regarding to log-likelihood in public (**S5A**) and in-house (**S5B**) real data analyses. Each color refers to a different initialization (total 10) of BREM-SC.

S6A

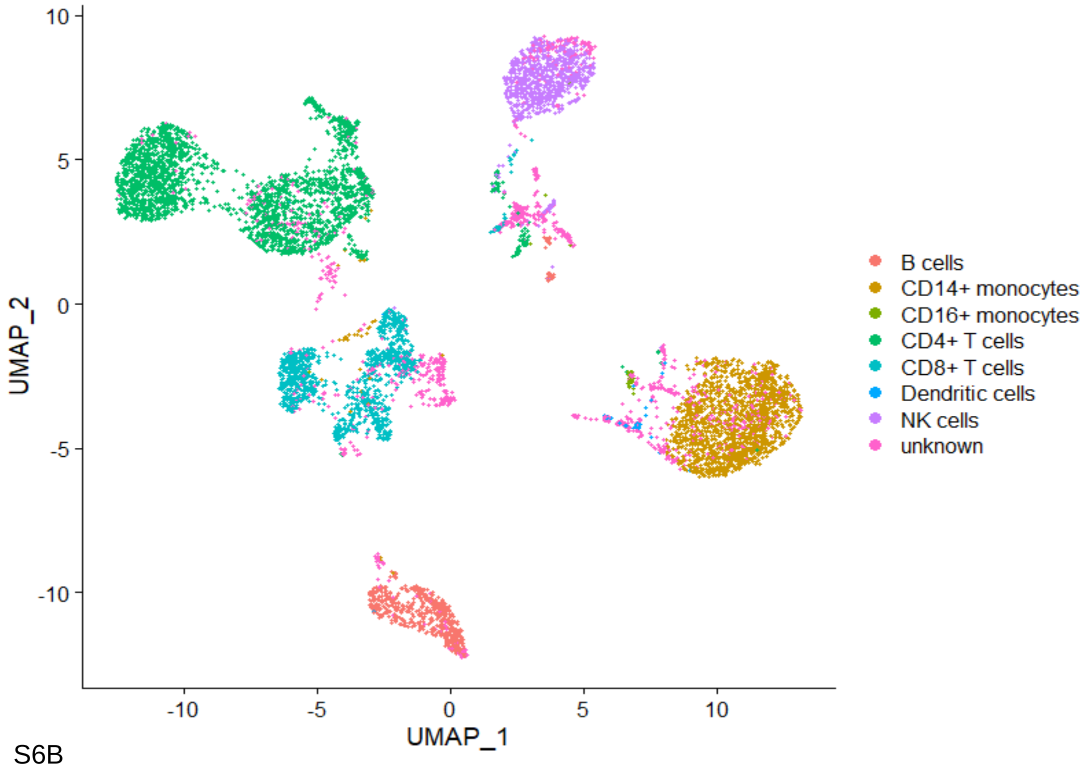


S6B

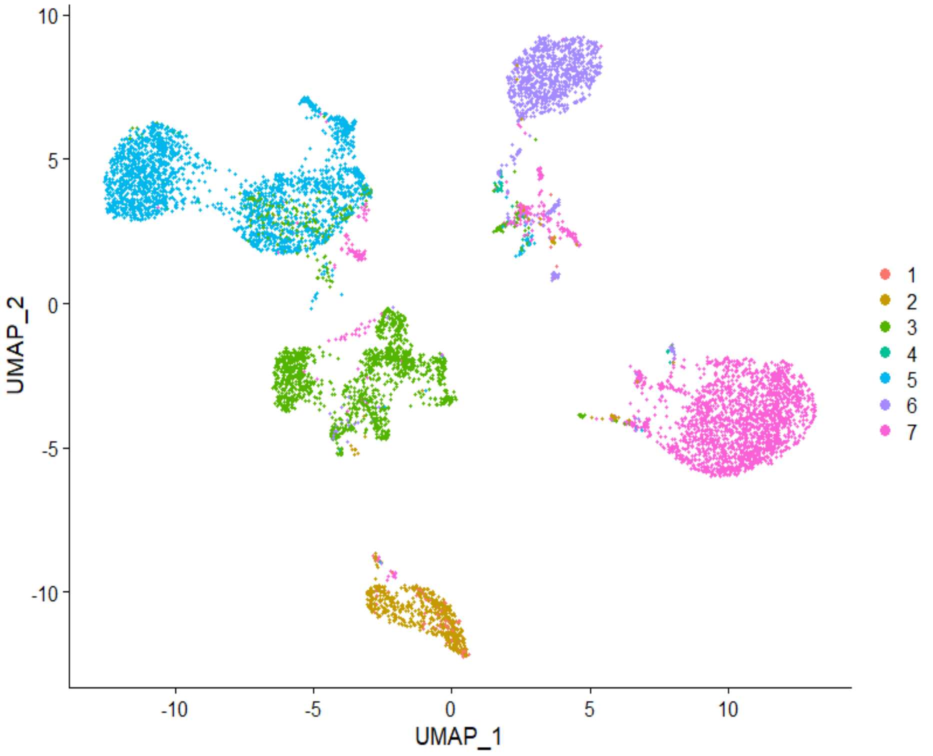


**Figure S6.** The performance of jointDIMM-SC for 10X public human PBMC CITE-Seq dataset. The UMAP projection of cells are colored by the ground truth (**S6A**) and jointDIMM-SC clustering results (**S6B**).

S7A

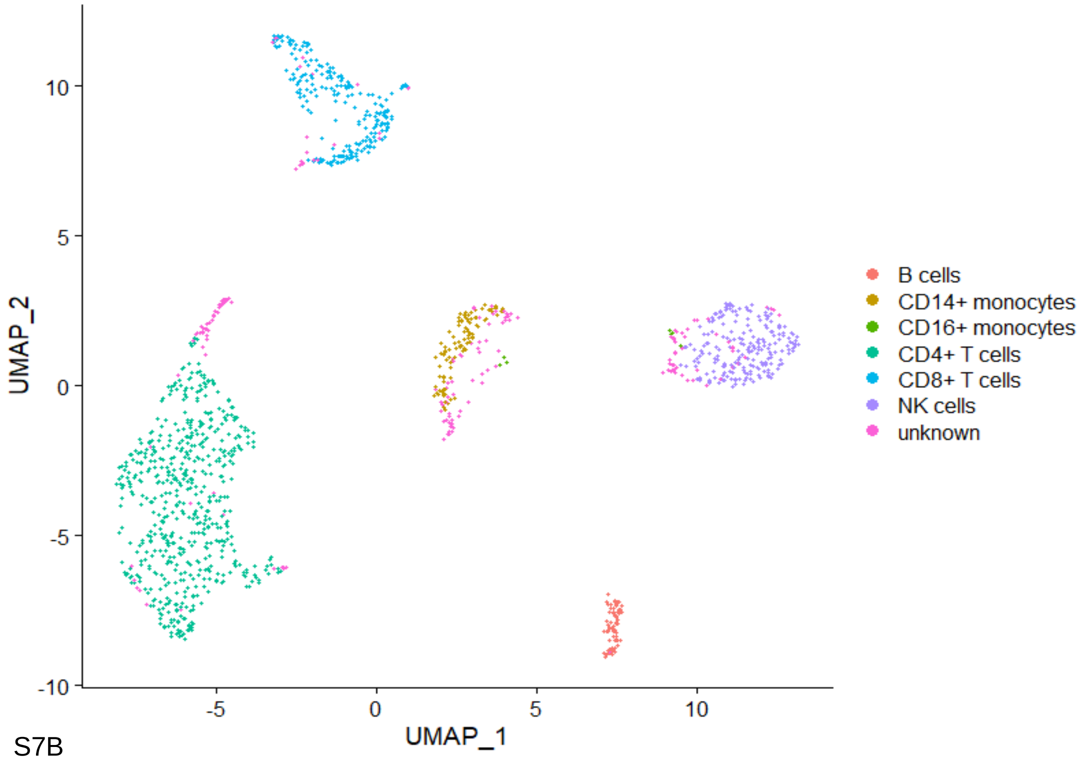


S7B

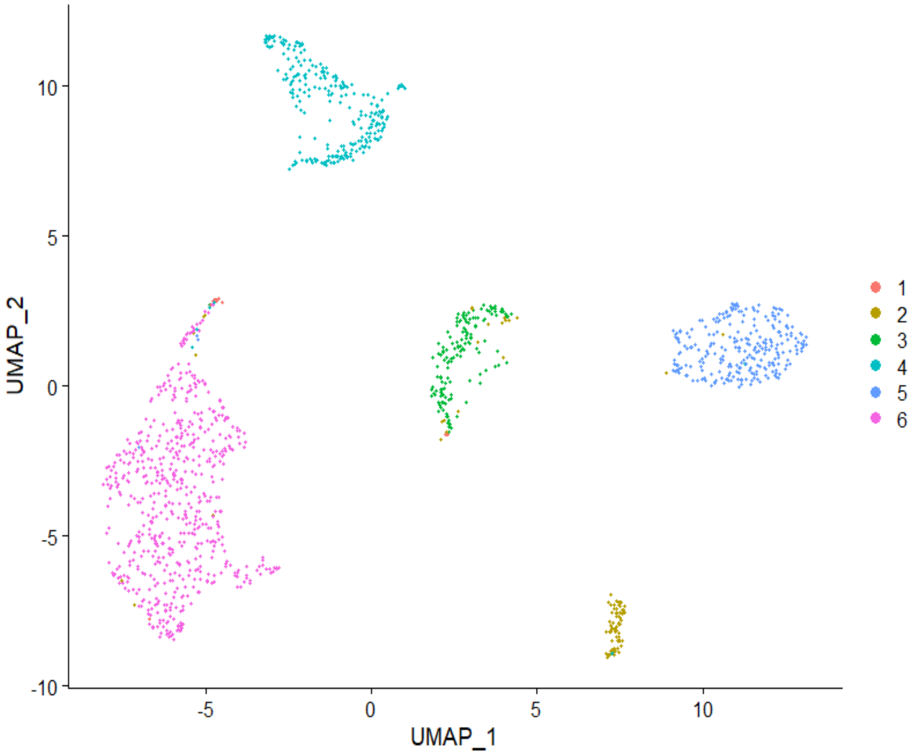


**Figure S7.** The performance of jointDIMM-SC for in-house human PBMC CITE-Seq dataset. The UMAP projection of cells are colored by the ground truth (**S7A**) and jointDIMM-SC clustering results (**S7B**).
